# Induction of bone marrow-derived cells myogenic identity by their interactions with the satellite cell niche

**DOI:** 10.1186/s13287-018-0993-z

**Published:** 2018-09-27

**Authors:** Kamil Kowalski, Matthieu Dos Santos, Pascal Maire, Maria A. Ciemerych, Edyta Brzoska

**Affiliations:** 1 Department of Cytology, Faculty of Biology, University of Warsaw, Miecznikowa 1 St, 02-096 Warsaw, Poland; 2 Institut Cochin, Université Paris-Descartes, Centre National de la Recherche Scientifique (CNRS), UMR 8104, Paris, France; 3 Institut National de la Santé et de la Recherche Médicale (INSERM) U1016, Paris, France

## Abstract

**Background:**

Skeletal muscle regeneration is possible thanks to unipotent stem cells, which are satellite cells connected to the myofibers. Populations of stem cells other than muscle-specific satellite cells are considered as sources of cells able to support skeletal muscle reconstruction. Among these are bone marrow-derived mesenchymal stem cells (BM-MSCs), which are multipotent, self-renewing stem cells present in the bone marrow stroma. Available data documenting the ability of BM-MSCs to undergo myogenic differentiation are not definitive. In the current work, we aimed to check if the satellite cell niche could impact the ability of bone marrow-derived cells to follow a myogenic program.

**Methods:**

We established a new in-vitro method for the coculture of bone marrow-derived cells (BMCs) that express CXCR4 (CXCR4^+^BMCs; the stromal-derived factor-1 (Sdf-1) receptor) with myofibers. Using various tests, we analyzed the myogenic identity of BMCs and their ability to fuse with myoblasts in vitro and in vivo.

**Results:**

We showed that Sdf-1 treatment increased the number of CXCR4^+^BMCs able to bind the myofiber and occupy the satellite cell niche. Moreover, interaction with myofibers induced the expression of myogenic regulatory factors (MRFs) in CXCR4^+^BMCs. CXCR4^+^BMCs, pretreated by the coculture with myofibers and Sdf-1, participated in myotube formation in vitro and also myofiber reconstruction in vivo. We also showed that Sdf-1 overexpression in vivo (in injured and regenerating muscles) supported the participation of CXCR4^+^BMCs in new myofiber formation.

**Conclusion:**

We showed that CXCR4^+^BMC interaction with myofibers (that is, within the satellite cell niche) induced CXCR4^+^BMC myogenic commitment. CXCR4^+^BMCs, pretreated using such a method of culture, were able to participate in skeletal muscle regeneration.

## Background

The bone marrow is a source of numerous cell populations. Among them are hematopoietic stem cells (HSCs) and bone marrow-derived mesenchymal stem cells (BM-MSCs). BM-MSCs are multipotent, self-renewing stem cells that are present in the mammalian bone marrow stroma [1–3]. They play a role in the growth and turnover of the bone and formation of the hematopoietic microenvironment [1–3]. In the mouse, subcutaneously transplanted BM-MSCs form bone and bone marrow that can be colonized by host epithelium and hematopoietic cells [4–7]. Moreover, it was shown that a single BM-MSC can give rise to osteogenic-, chondrogenic-, and adipogenic-derived cells, demonstrating its multipotency [4, 8, 9]. The ability of BM-MSCs to self-renew their population in vivo after serial transplantation has also been documented [10]. Thus, BM-MSCs fulfill the strict criteria characterizing multipotent stem cells: the ability to self-renew and differentiate into several cell types both in vitro and in vivo.

The ability of BM-MSCs to manifest myogenic potential is still controversial [1]. Human CD146^+^BM-MSCs were shown to be unable to undergo myogenic differentiation when transplanted into heterotopic sites or in vitro cultured in differentiating medium, i.e., in the presence of horse serum [11]. Thus, it was concluded that BM-MSCs do not present naive myogenic potential. However, the myogenic identity of BM-MSCs could be induced in vitro by overexpression of Notch intracellular domain (NICD) [12], β-catenin [13], Pax3 [14], or coculture with myoblasts, as well as in vivo by transplantation into regenerating skeletal muscle [15–23].

Under physiological conditions, skeletal muscle regeneration is possible thanks to satellite cells, which are muscle-specific unipotent stem cells occupying the myofiber niche localized between the basal lamina sheet of extracellular matrix (ECM) and the myofiber plasma membrane [24, 25]. The satellite cells express M-cadherin and CD34 which play important role in adhesion to the myofiber [26–28], as well as integrin α7 and β1, dystroglycan that binds laminin present in the ECM [29, 30], and syndecan-3 and syndecan-4 that act as coreceptors for integrins [31]. One of the receptors that is critical for the maintenance of satellite cell quiescence is Notch [32, 33]. The lack of Notch signaling leads to spontaneous satellite cell differentiation [33]. Satellite cells, activated in the case of muscle damage, proliferate, migrate, and differentiate into myoblasts and then myocytes that fuse to form multinucleated myotubes and myofibers. As a result, damaged muscle becomes reconstructed [24, 25]. Importantly, some of the satellite cells do not form multinucleated myotubes but self-renew and return to quiescence, supplying a satellite cell pool [24]. Satellite cell activation and satellite cell-derived myoblast proliferation and differentiation depend on the precisely orchestrated expression of myogenic regulatory factors (MRFs) such as Myod1 and Myf5, and finally myogenin [34, 35]. Importantly, the satellite cells fate is determined by extrinsic factors present within the local environment, in other words in the satellite cell niche, which includes growth factors, cytokines, adhesion molecules, and ECM that is composed of collagen IV, collagen VI, laminin-2, laminin-4, fibronectin, entactin, perlecan, decorin, and other proteoglycans [36–39]. Such an environment is formed by various cells present in intact or regenerating muscle, such as vessel-associated cells, immune cells, fibroadipogenic progenitors (FAPs), fibroblasts, and myofibers [36]. The satellite cell niche changes drastically in the case of muscle injury [36–39]. First, muscle injury generates an inflammatory process that affects the integrity of the niche, but which is required to remove the damaged myofibers, induce satellite cell proliferation and differentiation, and finally to restore the muscle homeostasis [36, 37]. Besides immune cells, damaged myofibers, fibroblasts, endothelial cells, and FAPs also appear as a source of growth factors, cytokines, and ECM required for efficient muscle regeneration [36, 38].

In the present work, we focused on the bone marrow-derived cells (BMCs), which is the cell population that is isolated from bone marrow and contains the fraction of BM-MSCs. In our previous studies we showed that in-vitro cultured mouse CXCR4^+^BMCs are able, with low frequency, to fuse with C2C12 myoblasts [40]. We also proved that stromal-derived factor-1 (Sdf-1) treatment led to increased tetraspanin CD9 expression facilitating CXCR4^+^BMC fusion with C2C12 myoblasts [40]. Currently, we have tested the myogenic potential of a BMC subpopulation that expresses CXCR4, a receptor for Sdf-1 which is the chemokine belonging to the cytokine family that plays an important role in stem cell mobilization [41]. We have established a new coculture system in which CXCR4^+^BMCs are permitted to interact with myofibers that provide the niche for satellite cells. Using such a method we were able to analyze if the tested cells could colonize the satellite cell niche in vitro and if such interactions could induce their myogenic engagement.

## Methods

The animal studies were approved by the Local Ethics Committee No. 1 in Warsaw, Poland (permit number 591/2014 and 046/2016) and Ethical Paris Descartes Cmmittee, France (CEEA34.PM.073.12) according to the European Union Directive on the approximation in laws, regulations, and administrative provisions of the Member States regarding protection of animals used for experimental and scientific purpose [42, 43].

### Isolation of CXCR4^+^BMCs

A whole bone marrow cell population was obtained from the tibialis and femoris bone of 3-month-old male C57BI6N-lacZ mice expressing β-galactosidase (β-gal). Bone marrow cells were washed from bones using phosphate-buffered saline (PBS) and centrifuged in Histopaque 1077 gradient (Sigma-Aldrich) to remove erythrocytes. BMCs expressing CXCR4 (CXCR4^+^BMCs(β-gal^+^)) were then selected using magnetic columns with antibody against CXCR4 and rabbit secondary antibody conjugated with paramagnetic particles accordingly to the manufacturer’s instructions (Miltenyi Biotec).

### Single myofiber isolation

Gastrocnemius muscles were isolated from 3-month-old male C57BI6N mice according to the method of Rosenblatt et al. [44]. Briefly, mice were killed by cervical dislocation and the muscles were carefully isolated from tendon to tendon, divided into smaller parts, and digested in 0.2% collagenase type I (Sigma-Aldrich) in Dulbecco’s modified Eagle’s medium (DMEM) at 37 °C for 2 h. Single myofibers were separated using pipette tips with a capillary. Myofibers were washed in PBS and intact, long myofibers were selected. Each myofiber was carefully transferred into a single hanging drop of DMEM supplemented with 20% fetal bovine serum (FBS), 10% horse serum (HS), 0.5% chick embryo extract (CEE), and 1% penicillin/streptomycin localized on the cover of a Petri dish. The cover was turned upside down and placed on the base of the same Petri dish filled with PBS to prevent evaporation of the hanging drops, as previously described [45].

### Coculture of CXCR4^+^BMCs(β-gal^+^) and myofibers

A single myofiber was selected and transferred together with approximately 100 CXCR4^+^BMCs expressing β-gal (CXCR4^+^BMCs(β-gal^+^)) into a hanging drop of DMEM supplemented with 20% FBS, 10% HS, 0.5% CEE, and 1% penicillin/streptomycin. CXCR4^+^BMCs(β-gal^+^) were cocultured with the myofiber for 5 days in control or Sdf-1 (100 ng/ml)-supplemented medium.

### Coculture of fibroblasts and myofibers

Fibroblasts were isolated from the ears of 3-month-old male C57BI6N-lacZ mice expressing β-gal according to a standard protocol [46] and cultured in DMEM supplemented with 20% FBS, 10% HS, 0.5% CEE, and 1% penicillin/streptomycin, and passaged three times. Next, 100 fibroblasts and a single myofiber were cocultured for 5 days in a hanging drop of DMEM supplemented with 20% FBS, 10% HS, 0.5% CEE, and 1% penicillin/streptomycin. Fibroblasts were cultured in control or Sdf-1 (100 ng/ml)-supplemented medium.

### Analysis of CXCR4^+^BMCs(β-gal^+^) cocultured with myofibers in monolayer culture

After 5 days coculture of CXCR4^+^BMCs(β-gal^+^) with myofibers, they were transferred and washed three time in PBS to eliminate the cells that were not attached to the myofiber. Myofibers with attached CXCR4^+^BMCs(β-gal^+^) were then transferred to six-well dishes covered with Matrigel (Becton Dickinson) and cultured for 14 days in DMEM supplemented with 20% FBS, 10% HS, 0.5% CEE, and 1% penicillin/streptomycin. After 14 days, the cultures were analyzed using a Nikon Eclipse TE200 microscope equipped with Hoffman contrast.

### Detection of β-galactosidase activity

After 14 days of monolayer culture, the cells which migrated from myofibers were fixed with 0.5% glutaraldehyde (Sigma) for 10 min. Next, cells were washed in PBS, incubated in 1 mM MgCl_2_ for 5 min, and exposed to X-gal working solution (40 mg/ml X-gal, 5 mM K_3_Fe(CN)_6_, 5 mM K_4_Fe(CN)_6_•H_2_O, 2 mM MgCl_2_ in PBS) until a blue color developed. Three independent experiments were performed.

### Transplantation of CXCR4^+^BMCs (β-gal^+^) into regenerating muscle

The gastrocnemius muscles of 3-month-old male C57BI6N mice were injured by injection of 25 μl 0.2 mM cardiotoxin (ctx; Latoxan) into both muscles. After 3 days of regeneration, CXCR4^+^BMCs(β-gal^+^) were transplanted into the muscles. The pretreated CXCR4^+^BMCs(β-gal^+^) cocultured with myofibers and Sdf-1 and control CXCR4^+^BMCs(β-gal^+^), in other words freshly isolated from bone marrow, were injected into regenerating muscles. Each muscle received 100,000 cells.

### Transplantation of CXCR4^+^BMCs(β-gal^+^) into regenerating muscles electroporated with Sdf-1

The tibialis anterior of 3-month-old male C57BI6N mice were injured by injection of 35 μl 12 μM ctx (Latoxan) into the muscles of both legs. After 4 days of ctx-induced regeneration, muscles were electroporated with a NEPA21 electroporator (Nepagene). The plasmids used were pUNO1 encoding mouse CXCL12 (SDF1) gene, isoform 1 (INVIVO gene), and control plasmid (H2B-RFP). Nine micrograms of Sdf-1 plasmid and 1 μg of H2B-RFP plasmid in 50 μl of PBS or 10 μg of H2B-RFP plasmid in 50 μl of PBS were injected into injured muscles. Then, after 24 h, CXCR4^+^BMCs(β-gal^+^) pretreated by coculturing with myofibers in the presence of Sdf-1 and control CXCR4^+^BMCs(β-gal^+^), in other words freshly isolated from bone marrow and sorted, were transplanted into regenerating and electroporated muscles. Each muscle received 100,000 cells in 50 μl of PBS. Next, muscles were analyzed 12 days postinjury (7 days after cells injection and 8 days after plasmid electroporation).

### Immunoanalysis of CXCR4^+^BMCs(β-gal^+^) and myofibers

Selected antigens were immunolocalized in: 1) freshly isolated CXCR4^+^BMCs(β-gal^+^) that were incubated for 30 min in DMEM supplemented with 20% FBS and allowed to attach to cover slides coated with poly-l-lysine; 2) CXCR4^+^BMCs(β-gal^+^) that were cultured for 24 h on Matrigel in DMEM supplemented with 20% FBS, 10% HS, 0.5% CEE, and 1% penicillin/streptomycin; and 3) single myofibers with attached CXCR4^+^BMCs(β-gal^+^) that were cultured for 5 days in hanging drops. The specimens were washed three times in PBS and fixed in 3% paraformaldehyde in PBS for 10 min. Next, specimens were washed with PBS, permeabilized with 0.05% Triton X-100 (Sigma-Aldrich) in PBS for 3 min. Additionally, myofibers with attached CXCR4^+^BMCs(β-gal^+^) were incubated with 15 mM NH_4_Cl for 20 min, followed by incubation with 10% HS (Life Technologies at room temperature for 30 min. Then, specimens were incubated in 0.25% glycine (Sigma-Aldrich) in PBS for 20 min, followed by incubation in 3% bovine serum albumin (BSA) in PBS (Sigma-Aldrich) containing 2% donkey serum (Sigma-Aldrich) at room temperature for 1 h. Next, specimens were incubated with primary antibodies (anti-CXCR4, Abcam; anti-nestin, Abcam; anti-CD45, Santa Cruz; anti-β-galactosidase, Abcam; anti-M-cadherin, Abcam; anti-syndecan-4, Abcam; anti-integrin-α7, Abcam; anti-Ki67, Abcam; anti-Myf5, Abcam; anti-Myod1, Abcam; and anti-NICD, Cell Signalling) diluted either in 3% BSA in PBS 1:100 (BMCs) or 5% HS/PBS 1:50 (myofibers with attached CXCR4^+^BMCs(β-gal^+^)) at 4 °C overnight, followed by incubation with appropriate secondary antibodies conjugated with Alexa Fluor 488 or 594 (Life Technologies) diluted either in 1.5% BSA/PBS (1:200) or 5% HS/PBS (1:100) at room temperature for 1–2 h. Nuclei were visualized with DraQ5 (Biostatus Limited) diluted in PBS (1:1000; specimens were incubated at room temperature for 5 min). The specimens were mounted with Fluorescent Mounting Medium (Dako Cytomation). The appropriate controls for secondary antibodies were used. The proportion of cells expressing CXCR4, nestin, or CD45 was counted on slides from 20 fields of view. The proportion of cells expressing M-cadherin, syndecan-4, integrin α7, Myf5, Myod1, Ki67, and NICD was counted on 10 myofibers during each experiment. Each analysis was repeated three times.

### Immunoanalysis of muscle sections

Seven or 21 days after CXCR4^+^BMCs(β-gal^+^) transplantation, gastrocnemius or tibialis anterior muscles were isolated and frozen in isopentane cooled with liquid nitrogen, transferred to −80 °C, cut into 7-μm thick sections using a cryomicrotome (Microm HM525NX), and processed for immunolocalization of selected antigens. Muscle sections were hydrated in PBS and fixed in 3% paraformaldehyde for 10 min. Next, samples were washed with PBS, permeabilized with 0.05% Triton X-100 (Sigma-Aldrich) in PBS, incubated in 0.25% glycine (Sigma-Aldrich) in PBS, followed by incubation in 3% BSA in PBS (Sigma-Aldrich) containing 2% donkey serum (Sigma-Aldrich) at room temperature for 1 h. Next, all samples were incubated with primary antibodies (anti-β-galactosidase, Abcam; and anti-laminin, Sigma Aldrich) diluted in 3% BSA in PBS (1:100) at 4 °C overnight, followed by incubation with secondary antibodies conjugated with Alexa Fluor 488 or 594 (Life Technologies) diluted in 1.5% BSA/PBS (1:200) at room temperature for 2 h. Nuclei were visualized with DraQ5 (Biostatus Limited) diluted in PBS (1:1000; samples were incubated at room temperature for 5 min). Samples were mounted with Fluorescent Mounting Medium (Dako Cytomation). The newly formed myofibers with centrally located nuclei were counted 7 days after cell administration. The number of all myofibers was counted 21 days after cells administration. Ten fields of view from 10 muscles were analyzed for each experiment.

### Enzyme-linked immunosorbent assay (ELISA)

Tibialis anterior muscles transfected either with control plasmid (H2B-RFP) or the that encoding Sdf-1 were isolated 12 days following injury (i.e. 7 days after cell injection and 8 days after plasmid electroporation). Muscles were homogenized in cOmplete™ Lysis-M EDTA-free (Roche). Sdf-1 level were measured using ELISA (R&D) according to the manufacturer’s protocol. The assay was conducted using a μQuant instrument (Biotek Instruments).

### Statistical analysis

Data are presented as mean ± standard deviation. Student’s *t* test was used for statistical analyses to compare each condition against the control, and *p* < 0.05 was considered significant.

## Results

### BMCs can colonize the satellite cell niche in vitro

**Fig. 1:**
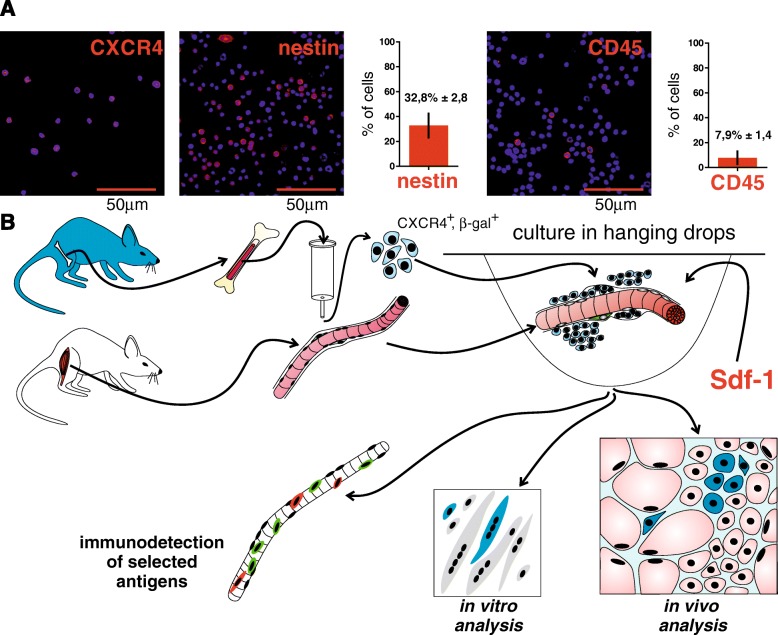
CXCR4^+^BMCs(β-gal^+^) isolation and experimental design. **a** Localization of CXCR4, nestin, and CD45 in a freshly isolated and sorted for CXCR4^+^ and β-gal^+^ subpopulation of BMCs; the proportion of cells expressing CXCR4, nestin, and CD45 are shown (*n* = 20). **b** Experimental design, description within the text. β-gal β-galactosidase, Sdf-1 stromal-derived factor-1

First, we isolated and characterized the CXCR4^+^BMCs and showed that a significant proportion of these cells expressed nestin (32.8 ± 2.8%) and only a small fraction expressed CD45 (7.9 ± 1.4%) (Fig. 1a). On the basis of nestin expression, we concluded that the CXCR4^+^BMC population contained a high percentage of BM-MSCs, that is cells that fulfill the criteria for multipotent mesenchymal stem cells [47].

To test whether CXCR4^+^BMCs are able to colonize the satellite cell niche we isolated them from the bone marrow of mice expressing β-galactosidase (β-gal^+^) and cocultured in hanging drops of medium with myofibers isolated from muscles of wild-type mice (Fig. 1b). As shown previously, the myofiber culture in the hanging drops allows the satellite cells to interact with their niche for a prolonged time and prevents their activation in vitro [45]. Thus, we assumed that such a culture method might be suitable to study the interactions of stem cells with myofibers and satellite cell niche. The expression of β-galactosidase allowed us to distinguish CXCR4^+^BMCs(β-gal^+^) from wild-type satellite cells connected with myofibers. Moreover, myofibers and cells were cultured in the presence or absence of Sdf-1. Such an approach allowed us to define the influence of Sdf-1 on the ability of CXCR4^+^BMCs(β-gal^+^) to colonize the satellite cell niche.

**Fig. 2:**
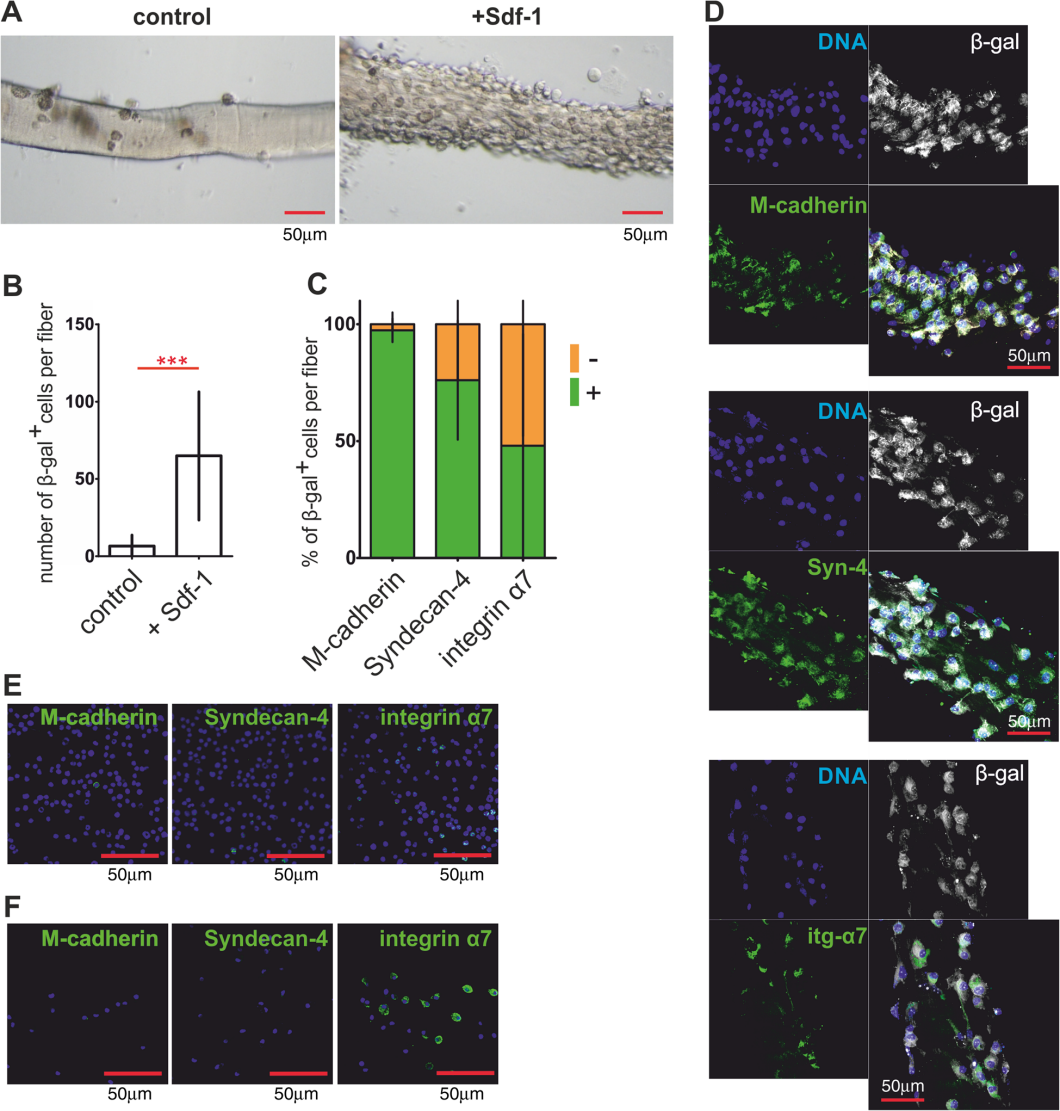
Characteristics of CXCR4^+^BMCs(β-gal^+^) cocultured with myofibers in hanging drops and analyzed after 5 days of culture. **a** Myofibers cocultured with CXCR4^+^BMCs(β-gal^+^) in control and Sdf-1-supplemented medium. **b** Proportion of CXCR4^+^BMCs(β-gal^+^) attached to myofibers, cultured in control and Sdf-1-supplemented medium (*n* = 30). **c** Percentage of CXCR4^+^BMCs(β-gal^+^) expressing M-cadherin, syndecan-4, and integrin-α7 attached to myofibers, cultured in the medium supplemented with Sdf-1 (*n* = 30). **d** Localization of M-cadherin, syndecan-4 (Syn-4), and integrin-α7 (itg-α7) in CXCR4^+^BMCs(β-gal^+^) cocultured with myofibers in medium supplemented with Sdf-1. **e** Localization of M-cadherin, syndecan-4 and integrin-α7 in freshly isolated CXCR4^+^BMCs(β-gal^+^). **f** Localization of M-cadherin, syndecan-4, and integrin-α7 in CXCR4^+^BMCs(β-gal^+^) cultured in monolayer on Matrigel and analyzed after 24 h of culture. ***p < 0.001. β-gal β-galactosidase

After 5 days of coculture, CXCR4^+^BMCs(β-gal^+^) were able to bind to the myofibers and this ability dramatically increased in the presence of Sdf-1 (Fig. 2a, b). At the same time, CXCR4^+^BMCs(β-gal^+^) that attached to the myofibers started to express the adhesion proteins M-cadherin, syndecan-4, and integrin α7 characteristic for satellite cells (Fig. 2c, d). The proportion of β-gal^+^ cells expressing integrin α7 differed between each analyzed fiber and varied from 10% to 100%.

To show that expression of adhesion proteins resulted from cell interaction with the myofiber we analyzed freshly isolated CXCR4^+^BMCs(β-gal^+^) (Fig. 2e) as well as those that were in-vitro cultured on Matrigel for 24 h (Fig. 2f). Both types of cells did not express M-cadherin and syndecan-4, but some of them expressed integrin α7 (Fig. 2e, f). We showed previously that Sdf-1 did not change the expression of adhesion proteins, except for CD9 [40]. Thus, we concluded that interaction of CXCR4^+^BMCs(β-gal^+^) with myofibers induced the expression of adhesion proteins characteristic for satellite cells. Moreover, Sdf-1 significantly increased the number of CXCR4^+^BMCs(β-gal^+^) able to bind myofibers.

### Interaction of CXCR4^+^BMCs(β-gal^+^) with myofibers induces their myogenic differentiation and leads to Notch activation

**Fig. 3:**
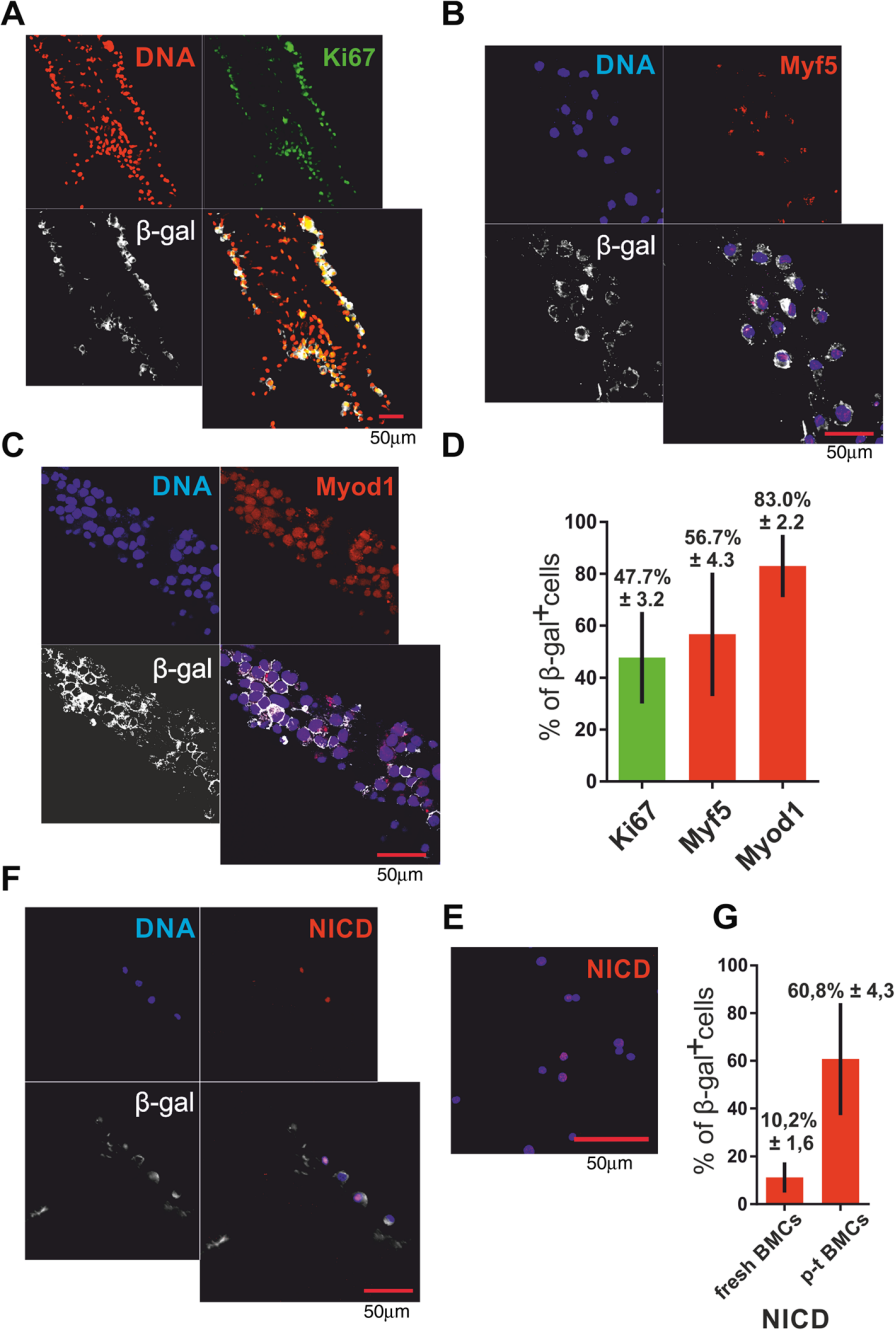
Expression of myogenic regulatory factors, Ki67, and NICD in CXCR4^+^BMCs(β-gal^+^) cocultured with myofibers for 5 days. **a** Localization of Ki67. **b** Localization of Myf5. **c** Localization of Myod1. **d** Percentage of CXCR4^+^BMCs(β-gal^+^) expressing Ki67, Myf5, and Myod1 attached to myofibers (*n* = 30). **e** Localization of Notch intracellular domain (NICD). **f** Localization of NICD in freshly isolated CXCR4^+^BMCs(β-gal^+^). **g** Percentage of CXCR4^+^BMCs(β-gal^+^) expressing NICD among cells freshly isolated from bone marrow-derived cells (fresh BMCs; *n* = 20) and after coculture with myofibers (p-t BMCs; *n* = 30). β-gal β-galactosidase

Knowing that in-vitro cultured CXCR4^+^BMCs(β-gal^+^) can interact with myofibers, we tested whether these cells could initiate a myogenic program. After 5 days of coculture, 47.7% of CXCR4^+^BMCs(β-gal^+^) associated with myofibers expressed Ki67 (in other words they proliferated; Fig. 3a, b). As we have previously documented, Sdf-1 did not influence proliferation of cells [48]. After 5 days of coculture, 56.7% of CXCR4^+^BMCs(β-gal^+^) expressed Myf5 and 83% expressed Myod1 (Fig. 3c, d). Thus, the large proportion of CXCR4^+^BMCs(β-gal^+^) underwent a myogenic commitment as the result of their interaction with myofibers. Sdf-1 did not impact the expression of Myf5 and Myod1. Thus, Sdf-1 stimulated binding of CXCR4^+^BMCs(β-gal^+^) to myofibers but did not change the proportion of cells expressing MRFs. Thus, we showed that our model allows induced expression of MRFs in CXCR4^+^BMCs(β-gal^+^). Next, we showed that CXCR4^+^BMCs(β-gal^+^) binding to myofiber was accompanied by activation of the Notch pathway. After 5 days of coculture, over 60% of CXCR4^+^BMCs(β-gal^+^) presented nuclear localization of NICD, the active intracellular Notch domain released after its activation (Fig. 3e–g). Importantly, only 10.2 ± 1.6% of freshly isolated CXCR4^+^BMCs(β-gal^+^) showed such localization of NICD (Fig. 3e–g). Thus, we conclude that binding of CXCR4^+^BMCs(β-gal^+^) to myofibers results in activation of the Notch signaling pathway.

**Fig. 4:**
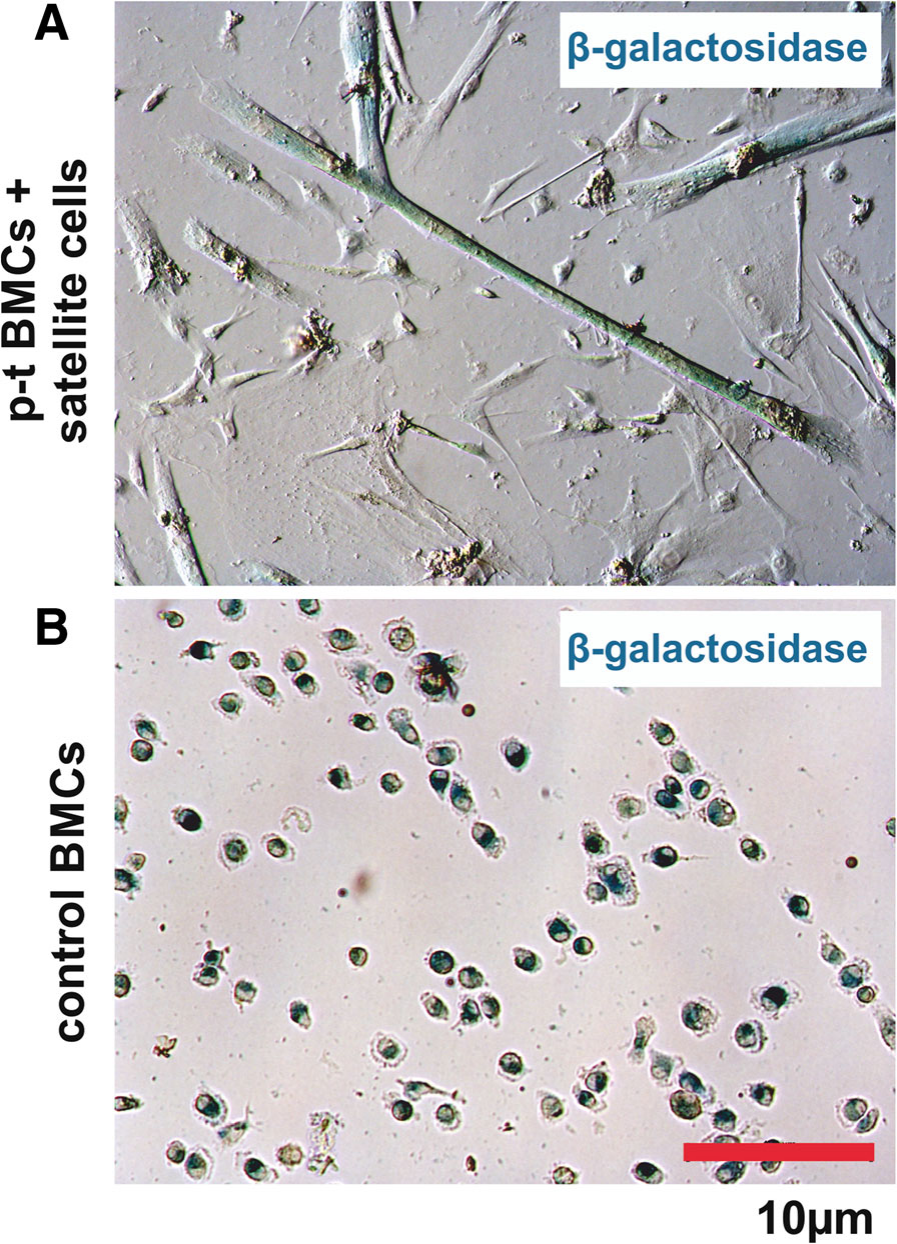
CXCR4^+^BMCs(β-gal^+^) cocultured with myofibers in monolayer on Matrigel. **a** CXCR4^+^BMCs(β-gal^+^) cocultured with myofibers in the presence of Sdf-1 for 5 days and transferred on Matrigel and cultured for 14 days. **b** Freshly isolated CXCR4^+^BMCs(β-gal^+^) cultured on Matrigel for 7 days. BMC bone marrow-derived cell, p-t pretreated

After 5 days of coculture in hanging drops myofibers with associated CXCR4^+^BMCs(β-gal^+^) were transferred into monolayer culture on Matrigel. After 2–3 days of such culture satellite cells that were associated with myofibers as well as CXCR4^+^BMCs(β-gal^+^) that colonized the satellite cell niche migrated from the surface of the myofibers and started to differentiate (Fig. 4). By detecting active β-galactosidase we were able to identify myotubes resulting from the fusion of cells derived from CXCR4^+^BMCs(β-gal^+^) and satellite cells (Fig. 4). Thus, we showed that CXCR4^+^BMCs(β-gal^+^), pretreated by culture on myofibers, could effectively fuse and form myotubes. Importantly, CXCR4^+^BMCs(β-gal^+^) that were freshly isolated from bone marrow did not fuse with each other or spontaneously form myotubes (Fig. 4).

**Fig. 5:**
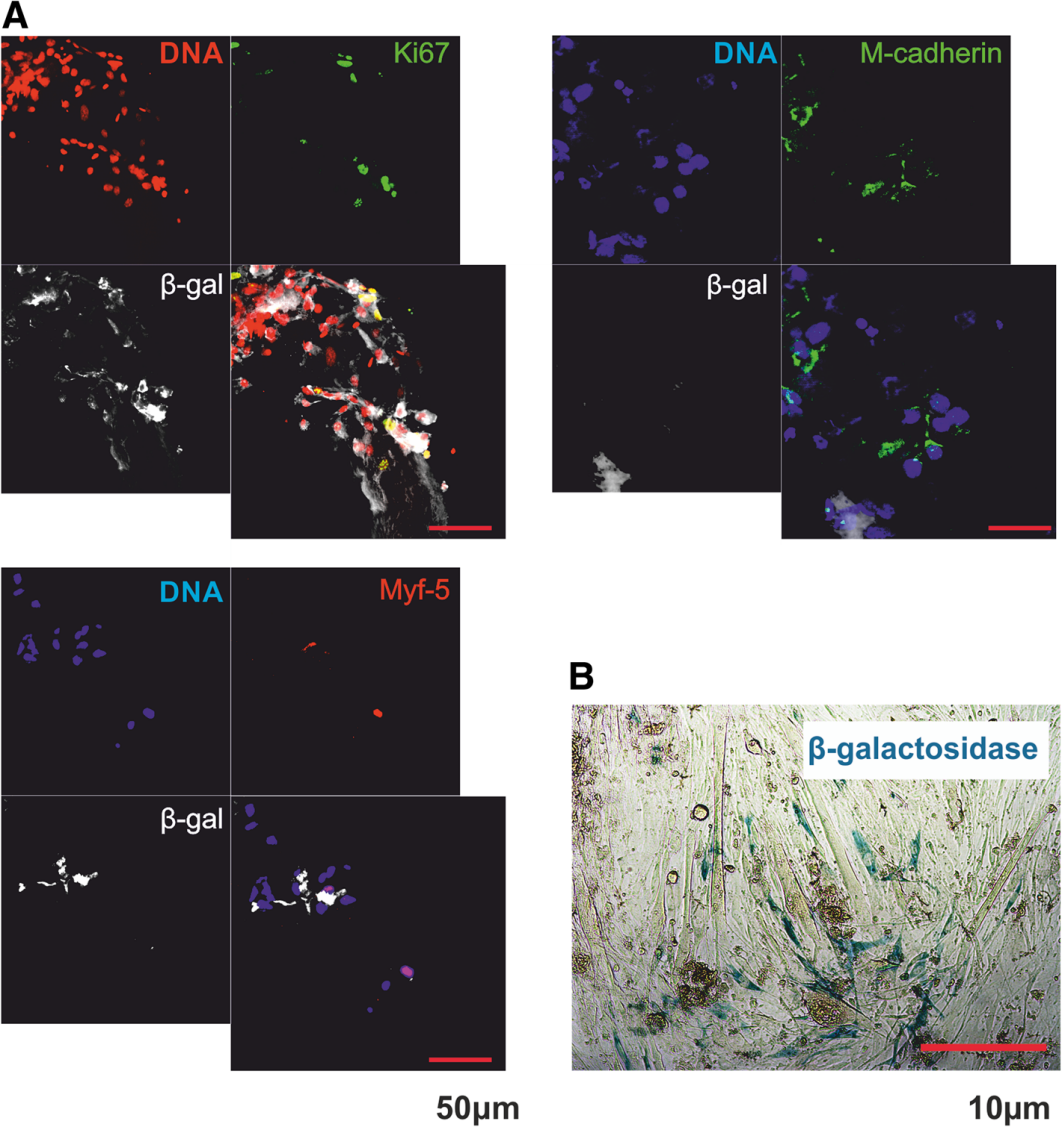
Analysis of β-gal^+^ fibroblasts cocultured with myofibers in hanging drops and transferred to monolayer on Matrigel. **a** Localization of Myf5, M-cadherin, and Ki67 in β-gal^+^ fibroblasts cocultured for 5 days with myofibers in hanging drops in medium supplemented with Sdf-1. **b** β-gal^+^ fibroblasts cocultured for 5 days with myofibers in hanging drops in medium supplemented with Sdf-1 and then transferred to monolayer culture on Matrigel and cultured for 14 days. β-gal β-galactosidase

We also examined whether cells other than the CXCR4^+^BMCs(β-gal^+^) can attach to myofibers and initiate a myogenic program. Thus, β-gal^+^ fibroblasts were cocultured with myofibers under the same conditions as those used for CXCR4^+^BMCs(β-gal^+^), including Sdf-1 treatment. The fibroblasts proliferated, as documented by the presence of Ki67 (Fig. 5a), but only a small proportion of them was able to bind to myofibers and colonize the satellite cell niche. Importantly, under such culture conditions, these cells never expressed MFRs (i.e., Myf5) or M-cadherin (Fig. 5a). Moreover, in monolayer culture on Matrigel where fibroblasts were released from myofibers, no multinucleated myotubes with active β-galactosidase was observed (Fig. 5b). Thus, we conclude that coculture with myofibers did not induce myogenic differentiation of fibroblasts.

### CXCR4^+^BMCs(β-gal^+^) pretreated by coculture on myofibers efficiently participate in skeletal muscle regeneration

**Fig. 6:**
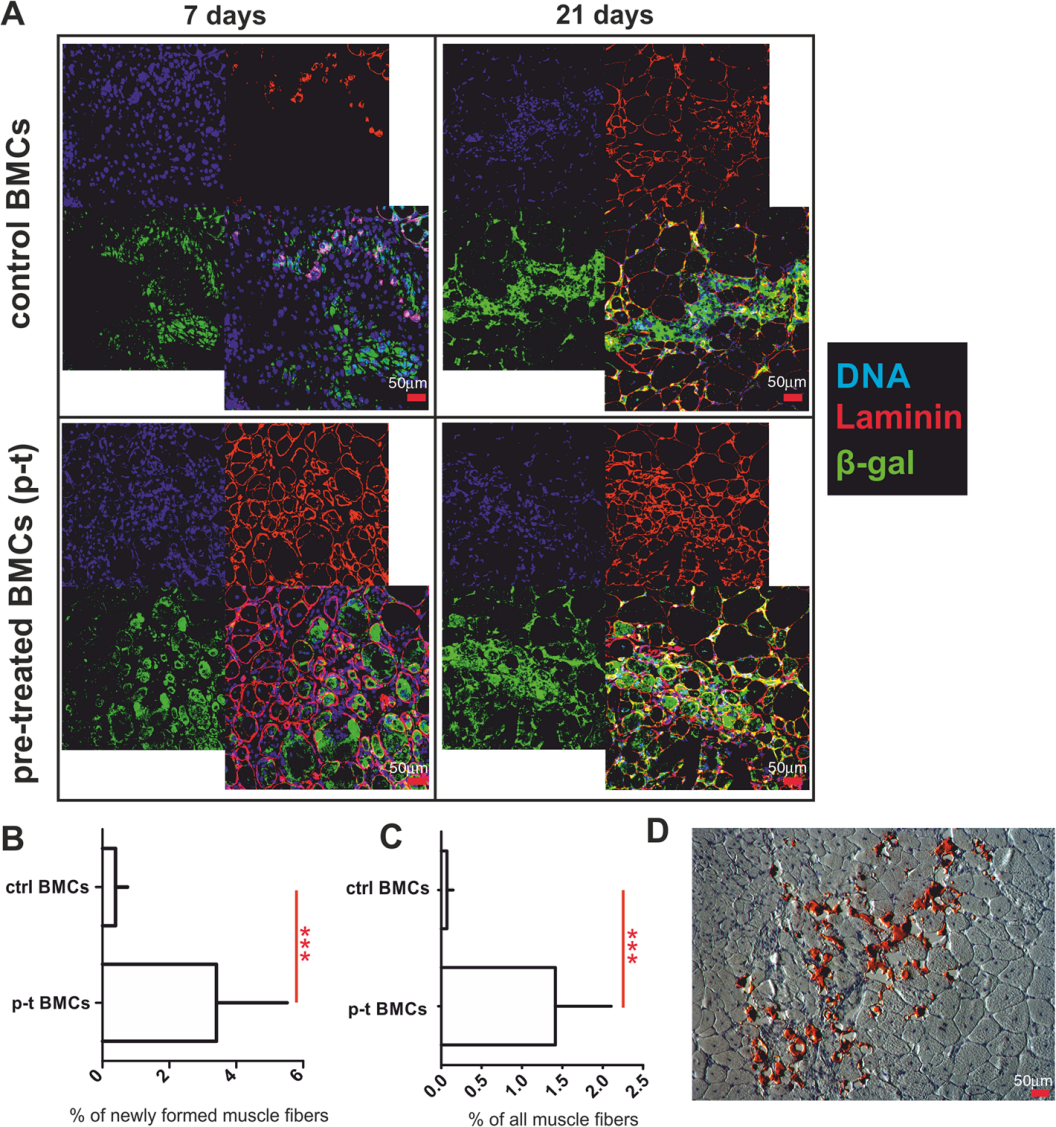
Analysis of regenerating muscles to which CXCR4^+^BMCs(β-gal^+^) were transplanted. **a** Localization of control, freshly isolated and sorted CXCR4^+^BMCs(β-gal^+^) (ctrl) and those pretreated (p-t) by coculture for 5 days in the presence of Sdf-1 with myofibers CXCR4^+^BMCs(β-gal^+^) in regenerating muscle after 7 and 21 days after cell administration. **b** Percentage of newly formed myofibers after 7 days and **c** of all myofibers expressing β-galactosidase after 21 days after cell administration (*n* = 10). **d** Oil Red O staining of adipocytes present in muscle sections after control CXCR4^+^BMCs(β-gal^+^) administration to regenerating muscle. ***p < 0.001. β-gal β-galactosidase

Since coculture with myofibers induces a myogenic identity of CXCR4^+^BMCs(β-gal^+^), we tested whether these cells possess increased ability to participate in skeletal muscle regeneration in vivo. Skeletal muscles of wild-type mice were injured with ctx and injected with CXCR4^+^BMCs(β-gal^+^) 3 days after injury. Two types of BMCs were tested: control CXCR4^+^BMCs(β-gal^+^) freshly isolated from bone marrow, and those pretreated by 5 days coculture with myofibers in the presence of Sdf-1. Regenerated muscles were analyzed 7 and 21 days after cell administration (Fig. 6). CXCR4^+^BMCs(β-gal^+^) were detected using immunolocalization of β-galactosidase. Control CXCR4^+^BMCs(β-gal^+^) participated in the formation of new myofibers with very low efficiency. Seven days after cell administration, only 0.4 ± 0.1% of new myofibers with centrally located nuclei expressed β-galactosidase (Fig. 6a, b). Twenty-one days after administration of cells, this proportion was even lower and had dropped to 0.07 ± 0.3% (Fig. 6a, c). Seven days after injection of CXCR4^+^BMCs(β-gal^+^) pretreated by coculture with myofibers in the presence of Sdf-1, 3.41 ± 0.7% of newly formed myofibers expressed β-galactosidase, and at day 21 as many as 1.4 ± 0.2% of all myofibers expressed β-galactosidase (Fig. 6a, b, c). Thus, after 7 and 21 days following cell administration, pretreated CXCR4^+^BMCs(β-gal^+^) resulted in the formation of 8.5- and 20-times more myofibers, respectively, compared with control cells (Fig. 6b, c). Thus, we have shown that coculture of CXCR4^+^BMCs(β-gal^+^) with myofibers increased their ability to participate in skeletal muscle regeneration. Interestingly, in muscles that received control CXCR4^+^BMCs(β-gal^+^), i.e., those freshly isolated from bone marrow, we observed numerous adipose cells, as shown by Oil Red O staining of skeletal muscles analyzed after 21 days following cells administration (Fig. 6d). This effect was observed in 4 out of 10 muscles analyzed. Importantly, we did not observe adipose cells accumulation in any of the 10 analyzed muscles injected with the CXCR4^+^BMCs(β-gal^+^) that were pretreated by coculture with myofibers.

**Fig. 7:**
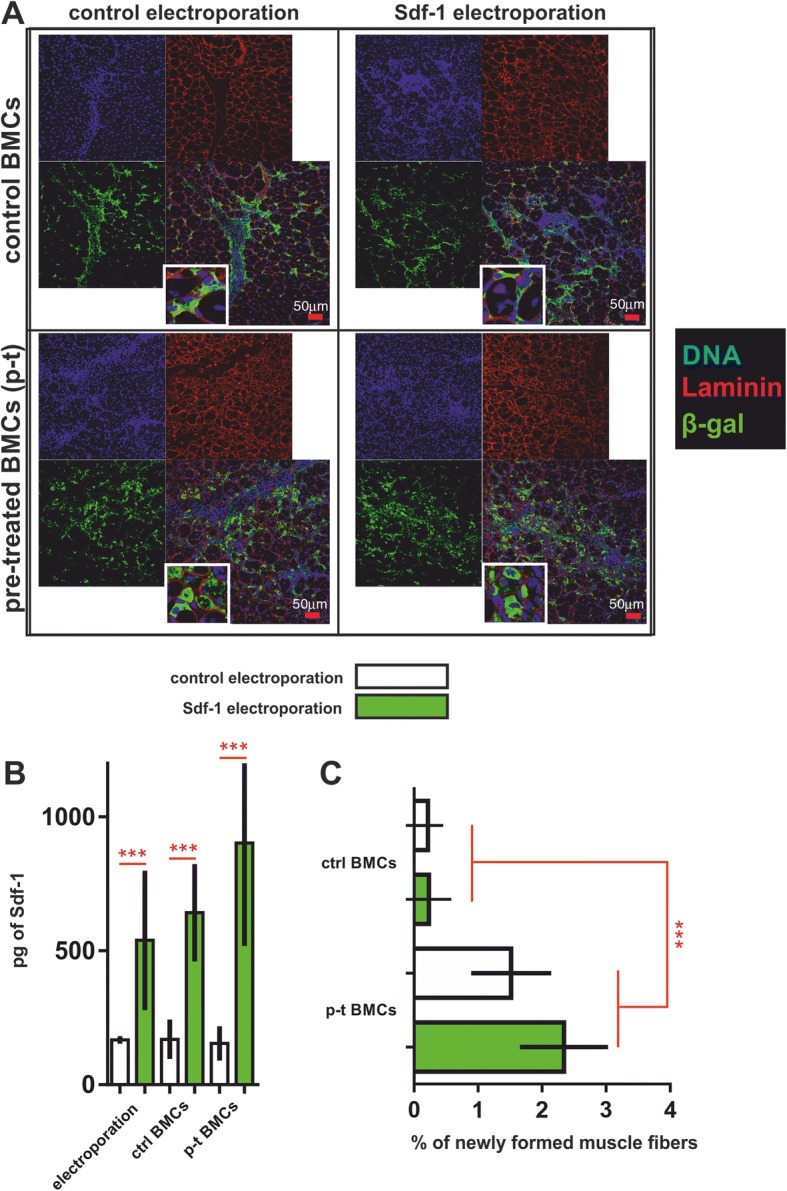
Analysis of regenerating muscles electroporated with either control plasmid (H2B-RFP) or plasmid coding Sdf-1 to which CXCR4^+^BMCs(β-gal^+^) were transplanted. Muscles were analyzed 12 days after injury, i.e., 7 days after cell injection and 8 days following electroporation. **a** Localization of transplanted cells; control, freshly isolated and sorted CXCR4^+^BMCs(β-gal^+^) (ctrl) or pretreated (p-t) by coculture for 5 days in the presence of Sdf-1 with myofibers CXCR4^+^BMCs(β-gal^+^) in regenerating muscle. **b** ELISA assessed Sdf-1 levels in skeletal muscles electroporated either with control or plasmid encoding Sdf-1 (*n* = 6). **c** Percentage of myofibers with the participation of CXCR4^+^BMCs(β-gal^+^) transplanted into muscles electroporated either with control or plasmid encoding Sdf-1 (*n* = 6). ***p < 0.001. β-gal β-galactosidase, BMC bone marrow-derived cell

To improve the homing of CXCR4^+^BMCs(β-gal^+^), we overexpressed Sdf-1 in regenerating muscle using an in-vivo electroporation system. Previously, we have shown that Sdf-1 injection improved skeletal muscle regeneration and stem cell mobilization [49, 50]. We also documented that pretreatment of cells with Sdf-1 induced migration of these cells after their transplantation into regenerating muscles [48]. Here, we tested whether the overexpression of Sdf-1 in injured and regenerating muscles would improve colonization of this tissue with transplanted cells. Thus, at day 4 of regeneration induced by ctx injection, muscles were electroporated either with control plasmid or with plasmid encoding Sdf-1 (Fig. 7). Twenty-four hours later the CXCR4^+^BMCs(β-gal^+^) were injected into the regenerating muscles. Introduction of Sdf-1-encoding plasmid resulted in a significant Sdf-1 increase within the muscle tissue (Fig. 7b). Twelve days postinjury (i.e., 7 days after cell injection and 8 days after electroporation), we localized transplanted CXCR4^+^BMCs(β-gal^+^). Control cells, in other words freshly isolated CXCR4^+^BMCs(β-gal^+^), were mostly found between myofibers and within connective tissue. Only some of them participated in the formation of myofibers (Fig. 7a, c). Transplantation of CXCR4^+^BMCs(β-gal^+^) that were pretreated by coculture with muscle fibers into electroporated muscles resulted in more abundant participation of these cells in the formation of new myofibers (Fig. 7a, c). Moreover, pretreated CXCR4^+^BMCs(β-gal^+^) formed more new myofibers in Sdf-1-overexpressing muscles. However, the difference between the muscles electroporated with control and Sdf-1-encoding plasmid was not statistically significant (Fig. 7c). Thus, we demonstrated that coculture of CXCR4^+^BMCs(β-gal^+^) with myofibers increased their ability to participate in skeletal muscle regeneration, and that Sdf-1 overexpression in muscles could support this process.

## Discussion

In our previous studies, we showed that Sdf-1 treatment allows skeletal muscle regeneration improvement via the mobilization of cells expressing CXCR4 and CD34 [49]. Importantly, the improvement of skeletal muscle regeneration stimulated by Sdf-1 and granulocyte colony-stimulating factor (G-CSF) was observed also in Pax7^−/−^ mice (i.e., mice lacking satellite cells), suggesting that stem cells other than satellite cells could be mobilized to injured muscles [50]. We also documented that Sdf-1 treatment of cells, such as satellite cell-derived myoblasts, CXCR4^+^BMCs, or embryonic stem cells, increased expression of tetraspanin CD9 that is crucial for myoblast adhesion and fusion [40]. As a result, Sdf-1-treated CXCR4^+^BMCs or embryonic stem cells fused more efficiently with myoblasts. Sdf-1 also altered cell migration and actin organization via focal adhesion kinase (FAK), cell division control protein 42 (Cdc42), and Ras-related C3 botulinum toxin substrate (Rac-1), as well as matrix metalloproteinase (MMP) activity [48, 49]. Currently, using a new in-vitro model, we were able to test the influence of the satellite cell niche on the differentiation of bone marrow-derived cells. We have shown that coculture of stem cells with myofibers could be used as a tool to study in-vitro interactions of exogenous stem cells with the satellite cell niche. Using a hanging drop coculture system, we showed that CXCR4^+^BMCs were able to colonize the satellite cell niche. Importantly, CXCR4^+^BMCs interacting with myofibers adopt a myogenic identity manifested by expression of MRFs and adhesion proteins characteristic of satellite cells, the ability to fuse with myoblasts, and to participate in skeletal muscle reconstruction. Sdf-1 treatment significantly increased this process. It has been documented previously that culture of myofibers in a hanging drop allowed satellite cells to retain their properties for prolonged time [45]. Such satellite cells maintained Pax7 expression as a result of their interactions with the niche [45]. Our results strongly suggest that the myofiber-specific niche can also influence cells other than satellite cells. We suggest that interaction with the satellite cell niche is an important step towards facilitating myogenic commitment of nonmyogenic cells.

The population of BMCs contains BM-MSCs that share the general features of other populations of MSCs and, importantly, meet the rigorous criteria of being multipotent stem cells: the ability to self-renew and to give rise to multilineage differentiation in vitro and in vivo [3]. In our study, we showed that a substantial proportion of the CXCR4^+^BMC population expressed nestin which is a marker of BM-MSCs [47], and only a small proportion of the CXCR4^+^BMCs were hematopoietic cells expressing CD45. Mouse BMCs expressing nestin are considered to be the “pure” population of BM-MSCs and fulfill all criteria for stem cells such as BM-MSCs [47] in that they are able to form colonies in in-vitro culture, undergo osteoblastic, adipogenic, and chondrocytic differentiation in vitro, and self-renew and expand after their serial transplantation. Furthermore, mouse BM-MSCs expressing nestin showed perivascular distribution in the bone marrow and colocalization with HSCs [47].

The ability of BM-MSCs to adopt a myogenic fate is still a matter of debate. It was clearly shown by Sacchetti and coworkers that human CD146^+^BM-MSCs are not able to undergo myogenic differentiation when transplanted into heterotopic sites or cultured in so-called differentiating medium, (i.e., in the presence of horse serum) [11]. Thus, CD146^+^BM-MSCs do not present a naive myogenic potential. Nevertheless, it is possible to induce the myogenic engagement of BM-MSCs (i.e., to reprogram them). Acquisition of the myogenic identity of the BMC adherent fraction could be induced in vitro by a DNA demethylating drug, for example 5-azacytidine [51]. However, human BM-MSCs very rarely form hybrid myotubes in coculture with C2C12 myoblasts (the proportion of hybrid myotubes was less than 0.038%) [11, 21]. Moreover, adherent rat BMCs constitutively expressing active β-catenin were able to fuse and form myotubes (fusion index of 27.1%) and expressed Pax7, Myod1, Myf5, myogenin, and MHC, as well as desmin, when cultured in the presence of 2% horse serum [13]. Pax3 overexpression in human and mouse adherent BMCs induced MRF expression and, as a result, these cells were able to fuse in the presence of horse serum [14, 52].

We showed here that activation of the Notch signaling pathway, manifested by nuclear localization of NICD, occurred in CXCR4^+^BMCs interacting with myofibers. Notch signaling is one of the major regulatory pathways that defines cell fate in relation to neighboring cells [35, 53]. As a result of CXCR4^+^BMC and myofiber interaction, these cells became competent to activate expression of MRFs. Notch signaling was also shown to regulate the myogenic potential of human and mouse mesoangioblasts which upregulated Notch at early stages of myogenic differentiation [54]. Decreased NICD levels caused by Notch1 and Dll1 knockdown resulted in impaired human and murine mesoangioblast myogenic differentiation and skeletal muscle engraftment after transplantation [54]. Dezawa and coworkers showed that overexpression of NICD and culture in horse serum induces myogenic differentiation of human and rat adherent BMCs resulting in their in-vitro and in-vivo participation in myofiber formation [12]. These cells expressed Pax7, Myod1, and myogenin, and importantly, fused with each other without the requirement for exogenous myoblasts [12]. Thus, the role of Notch signaling could differ between satellite cells and other cell types. Quiescent satellite cells express Notch-1, -2, and -3, and present high canonical Notch activity and the myofiber is the most likely source of Notch ligands [32, 33, 35]. Notch signaling inhibited pp38 and Myod1, early markers of activated satellite cells, and is silenced before the first satellite cell division [35, 55, 56]. Notch activation upregulates Pax7 and promotes the self-renewal of skeletal muscle satellite cells [57, 58]. Moreover, satellite cells or myoblasts cultured in the presence of Notch ligands exhibit a muscle stem-like state (i.e., high Pax7 and low Myod1 expression); however, their regenerative ability was lower than that of freshly isolated cells [53].

In our experimental model, CXCR4^+^BMCs cocultured with myofibers not only activated Notch but also increased the expression of adhesion proteins characteristic of satellite cells and MRFs characteristic of myoblasts. Importantly, such CXCR4^+^BMCs were functional, in other words they were able to fuse with myoblasts in vitro and in vivo. CXCR4^+^BMCs freshly isolated from bone marrow rarely participated in myofiber formation and after transplantation in adult muscle are found between myofibers or differentiate into adipocytes. We showed here that CXCR4^+^BMCs cocultured with myofibers in the presence of Sdf-1 more efficiently participate in skeletal muscle regeneration then control cells, i.e., those that were freshly isolated from bone marrow. Previously, Sacchetti and coworkers documented that human CD146^+^BM-MSCs failed to contribute to myofiber formation in regenerating skeletal muscles [11]. However, it was also described by many authors that adherent BMCs isolated from the whole population of bone marrow cells improved muscle regeneration [15], participated in the formation of new myofibers, and were able to occupy the satellite cell niche [16–23, 59]. However, it should be noted that the efficiency of BMC incorporation into new myofibers was very low. If BMCs were induced by NICD or Pax3 overexpression, the proportion of myofibers formed with their participation reached 11% or 16%, respectively [12, 14, 52].

## Conclusion

We observed that CXCR4^+^BMCs exposed to an environment specific for satellite cells (i.e., the satellite cell niche) were stimulated to adopt a myogenic fate (i.e., express MRFs and satellite cell-specific adhesion proteins). They were also able to fuse with myoblasts in vitro and more effectively participate in skeletal muscle regeneration, compared with control, freshly isolated from bone marrow, cells. Thus, we document that interaction with the specific niche could facilitate reprograming of multipotent stem cells. However, it should be noted that even after the myogenic commitment was induced in CXCR4^+^BMCs by their contact with the satellite cell niche, their ability to form new myofibers in regenerating muscle was still low. Thus, the use of mesenchymal stem cells in therapy is still characterized by many limitations [1, 3, 60, 61].

## Abbreviations

β-gal: β-galactosidase
BMC: Bone marrow-derived cell
BM-MSC: Bone marrow-derived mesenchymal stem cell
Cdc42: Cell division control protein 42
ctx: Cardiotoxin
CXCR4: CXC chemokine receptor 4
ECM: Extracellular matrix
FAK: Focal adhesion kinase
G-CSF: Granulocyte colony-stimulating factor
HSC: Hematopoietic stem cell
MMP: Matrix metalloproteinase
MRF: Myogenic regulatory factor
NICD: Notch intracellular domain
Rac-1: Ras-related C3 botulinum toxin substrate
Sdf-1: Stromal-derived factor-1
